# Supplementary material for: Content-rich biological network constructed by mining PubMed abstracts
Source: BMC Bioinformatics. 2004 Oct 8;5:147. doi: 10.1186/1471-2105-5-147 (PMC528731; doi:10.1186/1471-2105-5-147)
Supplement: Additional File 2 — The original results of the above study (non-essential files are deleted to keep the file size under the limit set by BMC bioinformatics). [file 1471-2105-5-147-S2.bz2 › chilibotAdditionalFile2/dip50/453ID10477718E10464/html/CCL2_CCR5.html]

 


 **CCL2** and **CCR5** 
  
Found 168 abstracts in PubMed, retrieved 50.  
 

 What does Google say? 
 PDF only 
| .edu only 

---

**Interactive relationship** (e.g. stimulation, inhibition, etc)

**Stimulatory relationship**- MIP 1alpha, MIP 1beta, RANTES, MCP  [ **CCL2** ]  2 and MCP  [ **CCL2** ]  4 were able to stimulate increases in intracellular calcium ions via activation of  **CCR5**  whereas MCP  [ **CCL2** ]  3 was without effect.  Ref: 11861332 Br J Pharmacol, 2002
- Also, increased expression of IFN gamma was associated with a significant increase in expression of RANTES and monocyte chemotactic protein 1  [ **CCL2** ] , chemokines that may inhibit HIV 1 infection by blocking viral attachment to CCR2 and  **CCR5**  co receptors.  Ref: 12048358 J Acquir Immune Defic Syndr, 2002
- The roles of  **CCR5**  in excitotoxic injury in  **CCR5**  deficient mice are compensated by increased CCR2 and CCR3 expression, which share the common MCP  [ **CCL2** ]  2 ligand with  **CCR5** .  Ref: 12807425 J Neurochem, 2003
**Neutral relationship**- Furthermore, MCP  [ **CCL2** ]  2 induces a robust, pertussis toxin sensitive calcium flux in primary lymphocytes, and cross desensitization studies indicate that MCP  [ **CCL2** ]  2 acts via  **CCR5** .  Ref: 11930329 J Infect Dis, 2002
- A number of inflammatory CC chemokines, including MIP 1 alpha, MIP 1 beta, RANTES, MCP  [ **CCL2** ]  2, and HCC 1 9 74 act as  **CCR5**  agonists, while MCP  [ **CCL2** ]  3 is a natural antagonist of the receptor.  Ref: 12402506 Receptors Channels, 2002
- **CCR5**  is a G protein coupled receptor for RANTES, MIP 1a, MIP 1b and MCP  [ **CCL2** ]  2 that functions as the front line coreceptor for human immunodeficiency virus type 1 infection.  Ref: 12837756 J Biol Chem< MedlineTA>J Biol Chem, 2002
- The present findings suggest that MCP 1  [ **CCL2** ] , RANTES, and IP 10 may participate in the pathogenesis of mercuric chloride induced tubulointerstitial fibrosis with mononuclear cell infiltration, via CCR2, CCR1 or  **CCR5** , and CXCR3, respectively.  Ref: 12834626 Exp Mol Pathol, 2003
- **CCR5**  is a G protein coupled receptor responding to four natural agonists, the chemokines RANTES regulated on activation normal T cell expressed and secreted, macrophage inflammatory protein MIP 1 alpha, MIP 1 beta, and monocyte chemotactic protein MCP  [ **CCL2** ]  2, and is the main co receptor for the macrophage tropic human immunodeficiency virus strains.  Ref: 12411445 J Biol Chem, 2003
- These data confirm that MCP  [ **CCL2** ]  2 is a ligand for  **CCR5**  on CD4 lymphocytes and can specifically block R5 HIV 1.  Ref: 11930329 J Infect Dis, 2002

**Non-interactive relationship** (e.g. studied together, co-existance, homology, etc.)

- Neuropathogenesis of lentiviral infection in macaques roles of CXCR4 and  **CCR5**  viruses and interleukin 4 in enhancing monocyte chemoattractant protein 1  [ **CCL2** ]  production in macrophages.  Ref: 12213709 Am J Pathol, 2002
- Only CpG ODN exacerbated HAF GN with an increase of glomerular macrophages, which was associated with massive albuminuria and increased renal MCP 1  [ **CCL2** ]   **CCL2** , RANTES CCL5, CCR1, CCR2, and  **CCR5**  mRNA expression.  Ref: 12538732 J Am Soc Nephrol, 2003
- RESULTS Expression of IP 10, Mig, I TAC, RANTES, CXCR3, and  **CCR5** , but not MCP 1  [ **CCL2** ]  and IL 8, increased significantly in both grade 2 and grade 3 rejection P< ; =0.0096.  Ref: 12544874 Transplantation, 2003
- Two hundred nine liver transplant recipients were genotyped using polymerase chain reaction sequence specific primers for the following polymorphisms RANTES 28, MCP 1  [ **CCL2** ]  2518, and  **CCR5**  59029.  Ref: 12462338 J Clin Immunol, 2002
- After day 7, a gradual increase in CCR2 and  **CCR5**  mRNA, monocyte chemoattractant protein MCP 1  [ **CCL2** ]  mRNA and protein expression was measured.  Ref: 11704636 Br J Pharmacol, 2001
- The presence of chemokine receptor  **CCR5** , CXCR3, CCR3 positive cells and chemokine MCP1  [ **CCL2** ] , MIP 1alpha, MIP 1beta, IP 10 positive cells in human periapical granulomas.  Ref: 11683586 Cytokine, 2001
- We investigated the effects of a number of naturally occurring chemokines MIP 1alpha, MIP 1beta, RANTES, MCP  [ **CCL2** ]  2, MCP  [ **CCL2** ]  3, MCP  [ **CCL2** ]  4 on different processes linked to the chemokine receptor  **CCR5**  in recombinant CHO cells expressing the receptor at different levels.  Ref: 11861332 Br J Pharmacol, 2002
- In conclusion, variants of RANTES 28, MCP 1  [ **CCL2** ]  2518, and  **CCR5**  59029 neither influenced the incidence of acute rejection nor affected long term allograft survival upon liver transplantation in the context of this analysis.  Ref: 12462338 J Clin Immunol, 2002
- TAK 799 N, N dimethyl N 4 2 4 methylphenyl dihydro 5H benzocyclohepten 8 yl carbonyl amino benzyl tetrahydro 2H pyran 4 aminium chloride, a dual CCR2  **CCR5**  antagonist, demonstrated high affinity for the rhesus CCR2B in competition with 125I hMCP 1 binding to the receptor K i =0.5nM and also potently blocked the MCP 1  [ **CCL2** ]  induced calcium mobilization mediated through the receptor.  Ref: 12826275 Biochem Pharmacol, 2003
- In parallel, CCR1 and  **CCR5**  expression is down regulated and CXCL8,  **CCL2** , CCL3 and CCL5 production is up regulated.  Ref: 12548563 Eur J Immunol, 2003
- In this study we show that  **CCL2**  monocyte chemotactic protein 1  [ **CCL2** ] , CCL5 RANTES, and CXCL12 stromal cell derived factor 1 enhanced IL 6 and IL 8 production by fibroblast like synoviocytes FLS from patients with RA, and their corresponding receptors, CCR2,  **CCR5** , and CXCR4, respectively, were expressed by RA FLS.  Ref: 11673556 J Immunol, 2001
- In addition, no association of RANTES 28, MCP 1  [ **CCL2** ]  2518, or  **CCR5**  59029 variants with long term liver graft survival was found.  Ref: 12462338 J Clin Immunol, 2002
- In parallel with down regulated SP and  **CCR5**  mRNA expression, cDNA array assays indicated that CP 9 45 treatment also inhibited IL 8 gene expression, while enhancing expression of fractalkine and monocyte chemotactic protein 3 MCP  [ **CCL2** ]  3.  Ref: 12098517 J Neuroimmunol, 2002
- Analysis of gene polymorphisms in the regulatory region of MCP 1  [ **CCL2** ] , RANTES, and  **CCR5**  in liver transplant recipients.  Ref: 12462338 J Clin Immunol, 2002
- The expression of interferon IFN gamma inducible protein IP 10, monokine induced by IFN gamma Mig, interferon inducible T cell alpha chemoattractant I TAC, regulated on activation normal T cell expressed and secreted RANTES, monocyte chemotactic protein MCP 1  [ **CCL2** ] , interleukin IL 8, and the receptors CXCR3 and  **CCR5**  were tested using quantitative, real time polymerase chain reaction.  Ref: 12544874 Transplantation, 2003
- The presence of chemokine MCP 1  [ **CCL2** ] , MIP 1alpha, MIP 1beta, IP 10, RANTES positive cells and chemokine receptor  **CCR5** , CXCR3 positive cells in inflamed human gingival tissues.  Ref: 12445801 Cytokine, 2002
- CONCLUSIONS This study suggested diagnostic as well as potential functional roles of the chemokine receptor pairs IP10 CXCR3, RANTES  **CCR5** , and MCP1  [ **CCL2** ]  CCR2 in rat models of transplant vasculopathy.  Ref: 12398874 J Heart Lung Transplant, 2002
- In J774 cells, CpG ODN induced the chemokines MCP 1  [ **CCL2** ]   **CCL2**  and RANTES CCL5 and the chemokine receptors CCR1 and  **CCR5** .  Ref: 12538732 J Am Soc Nephrol, 2003
- Recent studies have demonstrated that SMC possess a number of functional chemokine receptors, including  **CCR5** , CXCR4, and a receptor for monocyte chemoattractant protein 1  [ **CCL2** ]  MCP 1  [ **CCL2** ] .  Ref: 12851644 Microcirculation, 2003
- Whereas the amplitude of calcium mobilization induced by RANTES stimulation was lower in the  **CCR5**  CAM than the wild type WT receptor, MCP 1  [ **CCL2** ]  induced a higher signal in the CCR2 CAM than in CCR2 WT.  Ref: 12837756 J Biol Chem< MedlineTA>J Biol Chem, 2003
- The PBMC levels of  **CCR5**  ligands MIP 1 alpha beta and RANTES, and chemokines MCP 1  [ **CCL2** ] , IL 8, lymphotactin, IP 10 and I 309 were analysed by ribonuclease protection assay RPA.  Ref: 12021948 J Neurol, 2002
- At 26 weeks postirradiation, messages encoding the chemokines BLC now known as Scyb13, C10 now known as Scya6, IP 10 now known as Scyb10, MCP 1  [ **CCL2** ]  now known as Scya2  [ **CCL2** ] , MCP  [ **CCL2** ]  3 now known as Scya7, MIP 1gamma now known as Scya9, and RANTES now known as Scya5 and the chemokine receptors Ccr1, Ccr2,  **Ccr5**  and Ccr6 were elevated in fibrosis sensitive C57BL 6 mice.  Ref: 11839087 Radiat Res, 2002
- We investigated whether the functional polymorphisms of the chemokines RANTES, MCP 1  [ **CCL2** ]  and chemokine receptor  **CCR5**  are associated with the incidence of acute rejection and long term liver graft survival.  Ref: 12462338 J Clin Immunol, 2002
- In order to evaluate if these cells are infectabile by human immunodeficiency virus HIV and could be employed as a model in HIV research we exposed these cell lines to X4 T cell tropic and R5 macrophage tropic and subsequently tried to correlate their infectability with i level of chemokine coreceptor CXCR4 and  **CCR5**  expression, ii coreceptor functionality calcium flux, chemotaxis and phosphorylation of MAPK p42 44 and AKT and iii endogenous expression and secretion of HIV related chemokines which compete with the virus for binding to CXCR4 SDF 1 CXCL12 or  **CCR5**  MIP 1beta CCL4, MIP 1alpha CCL3, RANTES CCL5, MCP  [ **CCL2** ]  2 CCL8, MCP  [ **CCL2** ]  3 CCL7 and MCP  [ **CCL2** ]  4 CCL13.  Ref: 11737246 Eur J Haematol, 2001
